# The hidden cost of fitting in: social camouflage in adolescents with social communication disorder

**DOI:** 10.3389/fpsyg.2026.1839647

**Published:** 2026-06-05

**Authors:** Alireza Azizpour, Ghorban Hemati Alamdarloo, Setareh Shojaei, Shahram Moradi

**Affiliations:** 1 Department of Special Education, School of Education and Psychology, Shiraz University, Shiraz, Iran; 2 Research Group for Disability and Inclusion, Department of Health, Social and Welfare Studies, University of South-Eastern Norway, Porsgrunn, Norway; 3 Research Group for Health Promotion in Settings, Department of Health, Social and Welfare Studies, University of South-Eastern, Tønsberg, Norway

**Keywords:** adolescents, mental health, pragmatic language, social camouflaging, social communication disorder

## Abstract

**Introduction:**

This study compared social camouflaging in adolescents with and without social communication disorder (SCD) and examined whether camouflaging differedby gender.

**Methods:**

The sample comprised 120 adolescents aged 14–18 years from the Mashhad- Marghab region of Fars province, Iran, including 60 adolescents diag-nosed with SCD and 60 adolescents without SCD matched on age, gender, andeducational level. Social camouflaging was assessed using the CamouflagingAutistic Traits Questionnaire (CAT-Q), which captures compensation, masking,and assimilation. Data were analyzed using two-way analysis of variance andmultivariate analysis of variance.

**Results:**

The findings showed that adolescents withSCD reported significantly higher overall social camouflaging than adolescentswithout SCD. Male adolescents also reported higher camouflaging than femaleadolescents. At the subscale level, adolescents with SCD scored significantlyhigher on compensation, masking, and assimilation, and males scored higherthan females across all three subscales. These findings suggest that adolescentswith SCD may rely more heavily on camouflaging strategies to manage language-based social demands.

**Conclusion:**

Given growing evidence that camouflaging is associatedwith anxiety, depression, exhaustion, and identity strain in neurodevelopmentalpopulations, the findings are relevant to current work on language, emotions, andmental health. Greater clinical attention to the emotional burden of camouflagingin adolescents with SCD may help inform assessment and intervention.

## Introduction

Social communication disorder (SCD) is defined by enduring challenges in using verbal and non-verbal communication effectively in social contexts, including problems adapting language to context, following conversational rules, making inferences, and understanding implied meaning (American Psychiatric Association, 2013). These difficulties can interfere with peer relationships, classroom participation, and broader social functioning during adolescence, a developmental period in which belonging, friendship, and sensitivity to others’ evaluations become increasingly important (Blakemore and Choudhury, 2006; Brechwald and Prinstein, 2011). Emerging work also suggests that pragmatic communication difficulties rarely occur in isolation and are often associated with behavioural, academic, and emotional challenges (Saul et al., 2023). In that sense, SCD is highly relevant to current research at the intersection of language, emotion, and mental health.

One possible response to social-communication difficulties is social camouflaging. Camouflaging refers to strategies used to hide, compensate for, or minimize socially noticeable differences in everyday interactions (Hull et al., 2017). These strategies are commonly described within three overlapping domains: *compensation*, which involves developing alternative strategies to overcome social-communication challenges; *masking*, which refers to the suppression or concealment of behaviors perceived as socially atypical; and *assimilation*, which involves attempts to blend in with others by adopting socially normative behaviors (Hull et al., 2019).

Although camouflaging may help individuals manage immediate social demands, it is often cognitively and emotionally effortful and may carry psychological costs. A growing body of research has linked camouflaging to exhaustion, identity strain, reduced well-being, anxiety, depression, and suicidality, particularly among autistic individuals (Cage and Troxell-Whitman, 2019; Cassidy et al., 2020; Dighton and McAloon, 2026; Klein et al., 2025; Zhuang et al., 2023).

Most research on camouflaging has focused on autism rather than SCD specifically. However, the construct is also relevant to adolescents with SCD because their core difficulties concern the social use of language and the interpretation of social–emotional cues. Recent studies of autistic youth suggest that camouflaging begins early, is shaped by peer expectations, and may be experienced as stressful, confusing, and draining (Howe et al., 2023). Emerging evidence further indicates that camouflaging in autistic children and adolescents is associated with internalizing symptoms such as anxiety and depression, as well as externalizing difficulties, highlighting the potential mental health burden associated with sustained camouflaging behaviors (Dighton and McAloon, 2026). Quantitative work has further shown that autistic adolescents camouflage more than non-autistic adolescents in face-to-face contexts (Jedrzejewska and Dewey, 2022). Among socially anxious adolescents, camouflaging has also been linked to discrepancies in self- versus caregiver-reported anxiety and depressive symptoms (Lei et al., 2024). Together, these findings suggest that camouflaging is not only a social adaptation strategy but also a possible marker of emotional burden.

Despite these advances, there is very limited research on camouflaging in adolescents with SCD. This gap matters for at least two reasons. First, adolescents with SCD face persistent language-pragmatic demands in school and peer contexts, and these demands may encourage compensation or masking in ways that remain clinically under-recognized. Second, findings on gender differences in camouflaging remain mixed. Some studies have reported higher levels in females, others have reported higher levels in males, and some have found no meaningful difference (Hull et al., 2020; Milner et al., 2023; Ross et al., 2023). More evidence is therefore needed, especially in adolescent samples and in understudied cultural contexts.

Accordingly, the present study compared overall social camouflaging and its subdimensions (compensation, masking, and assimilation) in adolescents with and without SCD and examined whether camouflaging differed by gender. By focusing on a population defined by language-pragmatic difficulty rather than autism diagnosis alone, the study extends current discussion of camouflaging into a domain that speaks directly to verbal interaction, emotional adjustment, and mental health risk. Specifically, the study examined whether adolescents with and without SCD differed in overall social camouflaging and its subdimensions, and whether these differences varied by gender.

## Materials and methods

### Population, sample, and sampling method

The statistical population for this study included all adolescents aged 14–18 years with and without social communication disorder (SCD) in the Mashhad-Marghab region, Fars province, Iran. The sample consisted of 120 adolescents, including 60 adolescents with SCD and 60 adolescents without SCD. Both groups had identical gender distributions (16 females and 44 males).

Participants with SCD were recruited using a purposive sampling strategy. Secondary schools across the region were contacted, and school leaders (principals and vice-principals) were provided with a description of the DSM-5 criteria for social communication disorder (SCD) (American Psychiatric Association, 2013). They were asked to nominate students who might exhibit characteristics consistent with SCD.

Students identified through this process received information about the purpose of the clinical assessment and the research project. Participation in both the assessment and the study was entirely voluntary, and adolescents who agreed to participate were referred for clinical assessment by a licensed psychiatrist. Inclusion in the study required meeting DSM-5 diagnostic criteria for SCD. Diagnostic evaluations were based on comprehensive clinical assessments, including developmental history, pragmatic language functioning, and broader social communication abilities. Because DSM-5 criteria specify that SCD should not be diagnosed in the presence of autism spectrum disorder (ASD), the assessment process also considered broader patterns of restricted and repetitive behaviors and interests associated with ASD. Adolescents whose presentation was considered more consistent with ASD were not included in the SCD group. Although standardized structured diagnostic interviews (e.g., SCID) were not used, assessments followed established clinical practices in specialized services within the study region. A total of 60 adolescents met the inclusion criteria and were included in the SCD group.

Adolescents without SCD were recruited using a convenience sampling strategy, primarily through secondary schools that agreed to participate in the study. Potential participants were identified in collaboration with school staff, and students received information about the study prior to providing voluntary consent. This approach was adopted due to practical and logistical considerations and to facilitate matching with the SCD group on key demographic variables, including age, gender, and educational level.

No formal clinical screening or structured diagnostic interview (e.g., SCID) was conducted for the comparison group. In addition, psychiatric comorbidities were not systematically assessed or used as exclusion criteria in either group. This approach was intended to reflect the heterogeneity typically observed in general adolescent populations.

Group comparability was further examined across a range of sociodemographic variables. No statistically significant differences were found between adolescents with and without SCD in mean age (t-test), educational level, parental employment status, parental marital status (divorced or separated), parental death, or family income (chi-square tests), nor in family size (t-test). These findings indicate that, beyond the matching variables, the groups were broadly comparable in terms of key family and socioeconomic characteristics.

### Instrument

#### Camouflaging autistic traits questionnaire (CAT-Q)

The Camouflaging Autistic Traits Questionnaire (CAT-Q) was developed by Hull et al. (2019) to assess social camouflaging. It contains 25 items across three subscales: compensation (9 items), masking (8 items), and assimilation (8 items). Items are rated on a seven-point Likert scale from strongly disagree (1) to strongly agree (7). Total scores range from 25 to 175, with higher scores indicating greater camouflaging. Hull et al. (2019) reported excellent internal consistency for the total scale (alpha = 0.94) and for the subscales (compensation = 0.91, masking = 0.85, assimilation = 0.92), as well as good test–retest reliability (*r* = 0.77). Evidence supporting the validity of the scale has also been reported by Dell'Osso et al. (2022). In the present study, the CAT-Q demonstrated excellent internal consistency, with a Cronbach’s alpha coefficient of 0.973 for the total scale. Participants completed the paper-and-pencil version of the CAT-Q individually at school, which took approximately 5–10 min. Because the CAT-Q was originally developed in autistic samples, its use in adolescents with SCD should be interpreted as an assessment of camouflaging-like social adaptation strategies rather than as a disorder-specific diagnostic indicator.

#### Ethical considerations

Adolescents provided verbal consent to participate in the study. Before participation, they were informed about the purpose of the research, the voluntary nature of participation, and the confidentiality of the collected data. Participants were informed that they could withdraw from the study at any time without consequences. The study was approved by the ethical review board of the regional Special Education Organization.

## Results

Table 1 presents the total score of social camouflage of subjects based on adolescents’ group and adolescents’ gender.

**Table 1:** Means and standard deviations for total social camouflage scores by adolescent group and gender.

Adolescent group	Male, *M* (SD)	Female, *M* (SD)	Total, *M* (SD)
Adolescents with SCD	116.98 (27.06)	99.81 (34.33)	112.40 (29.87)
Adolescents without SCD	63.93 (8.74)	56.87 (7.85)	60.40 (8.97)
Total	95.47 (33.90)	71.80 (29.33)	86.40 (34.12)

SCD, social communication disorder; M, mean; SD, standard deviation.

A two-way ANOVA was conducted to compare mean social camouflaging scores as a function of adolescent group (with SCD vs. without SCD) and gender (male vs. female). Before running the analysis, Levene’s test was used to examine homogeneity of variances. Because Levene’s test was not significant [Levine Statistic = 20.738, *p* = 0.241], the assumption was considered satisfied and the ANOVA was performed. The results are presented in Table 2.

**Table 2:** Results of the two-way ANOVA for total social camouflage scores by adolescent group and gender.

Source	Sum of squares	df	Mean square	*F*	*p*	*η* ^2^
Adolescent group	60660.99	1	60660.99	132.34	0.000	0.527
Adolescent gender	3865.61	1	3865.61	8.43	0.003	0.072
Adolescent group × gender	671.34	1	671.34	1.47	0.230	0.007
Error	53170.75	116	458.37			
Total	1034292.00	120				

SCD, social communication disorder; F, univariate ANOVA *F* statistic; *p* = probability value.

The two-way ANOVA showed significant main effects of adolescent group and gender on overall social camouflaging. Adolescents with SCD reported significantly higher social camouflaging than adolescents without SCD [*F* = 132.34, *p* = 0.000, *η*^2^ = 0.527]. Male adolescents also reported significantly higher social camouflaging than female adolescents [*F* = 8.43, *p* = 0.003, *η*^2^ = 0.072].

Moreover, Table 3 presents the subscales score of social camouflage of subjects based on adolescents’ group and adolescents’ gender.

**Table 3:** Means and standard deviations for the subscales of social camouflage by adolescent group and gender.

Subscale	Adolescent group	Male, M (SD)	Female, M (SD)	Total, M (SD)
Compensation	Adolescents with SCD	40.95 (10.26)	36.50 (12.33)	39.77 (10.93)
	Adolescents without SCD	22.97 (3.61)	19.93 (2.70)	21.45 (3.51)
Masking	Adolescents with SCD	37.41 (8.84)	31.50 (10.91)	35.83 (9.70)
	Adolescents without SCD	20.73 (3.00)	18.17 (3.16)	19.45 (3.32)
Assimilation	Adolescents with SCD	38.36 (9.04)	31.75 (11.53)	36.60 (10.10)
	Adolescents without SCD	20.57 (3.02)	18.40 (2.88)	19.48 (3.13)

SCD, social communication disorder; M, mean; SD, standard deviation.

A MANOVA was used to compare the three subscales of social camouflaging as a function of adolescent group (with SCD vs. without SCD) and gender (male vs. female). Before conducting the MANOVA, Levene’s test was used to examine homogeneity of variances for compensation [Levine Statistic = 21.447, *p* = 0.162], masking [Levine Statistic = 18.012, *p* = 0.112], assimilation [Levine Statistic = 18.193, *p* = 0.120],and Box’s M test was used to examine homogeneity of covariance matrices [Box’s M test = 116.772, *p* = 0.149]. Neither test was significant, indicating that the assumptions for MANOVA were met. Wilks’ Lambda values are presented in Table 4.

**Table 4:** Wilks’ Lambda values from the multivariate ANOVA for the subscales of social camouflage by adolescent group and gender.

Source	Wilks’ Lambda	*F*	df	df error	*p*
Adolescent group	0.48	41.62	3	114	0.001
Adolescent gender	0.89	4.70	3	114	0.004
Adolescent group × gender	0.96	1.53	3	114	0.212

Wilks’ Lambda is the multivariate test statistic used in MANOVA. Smaller values indicate stronger group differences across the combined dependent variables. *F*, approximate *F* statistic; *p*, probability value.

As shown in Table 4, the multivariate effect of adolescent group on the combined dependent variables was significant, and the multivariate effect of gender was also significant. However, the interaction between adolescent group and gender was not significant at the multivariate level. To determine which dependent variables contributed to these effects, follow-up univariate ANOVAs were conducted; the results are presented in Table 5.

**Table 5:** Results of the MANOVA for the subscales of social camouflage.

Variable	Adolescent group *F*	Adolescent group *p*	*η* ^2^	Adolescent gender *F*	Adolescent gender *p*	*η* ^2^	Adolescent group × gender *F*	Adolescent group × gender *p*	*η* ^2^
Compensation	119.84	0.000	0.508	5.58	0.020	0.046	0.114	0.736	0.001
Masking	119.68	0.000	0.508	10.28	0.002	0.081	0.792	0.375	0.007
Assimilation	120.80	0.000	0.510	10.63	0.001	0.084	1.450	0.231	0.012

*F*, univariate ANOVA *F* statistic; *p*, probability value. These analyses were conducted as follow-up tests to the MANOVA.

The follow-up analyses showed significant effects of adolescent group on compensation [*F* = 119.84, *p* = 0.000, *η*^2^ = 0.508], masking [*F* = 119.68, *p* = 0.000, *η*^2^ = 0.508], and assimilation [*F* = 120.80, p = 0.000, *η*^2^ = 0.510]. Gender also had significant effects on compensation [*F* = 5.58, *p* = 0.020, *η*^2^ = 0.046], masking [*F* = 10.28, *p* = 0.002, *η*^2^ = 0.081], and assimilation [*F* = 10.63, *p* = 0.001, *η*^2^ = 0.084]. Overall, adolescents with SCD and male adolescents scored higher across all three dimensions of camouflaging. Finally, the interaction between adolescent group and gender was not significant for compensation [*F* = 0.114, *p* = 0.736, *η*^2^ = 0.001], masking [*F* = 0.375, *p* = 0.003, *η*^2^ = 0.007], or assimilation [*F* = 1.450, *p* = 0.231, *η*^2^ = 0.012].

## Discussion

The present study compared social camouflaging in adolescents with and without SCD and examined whether camouflaging differed by gender. The findings showed that adolescents with SCD reported higher overall camouflaging than adolescents without SCD, and that male adolescents reported higher camouflaging than female adolescents. The same pattern was observed for compensation, masking, and assimilation. These findings extend the literature by examining camouflaging in a group defined by language-pragmatic difficulty and by linking the construct more directly to questions of verbal interaction and emotional well-being.

The higher level of camouflaging among adolescents with SCD is theoretically plausible. SCD involves persistent difficulty using language effectively in social situations, including problems with conversational reciprocity, contextual adaptation, inference, and interpretation of non-verbal cues (American Psychiatric Association, 2013). In adolescence, when peer acceptance and social comparison become increasingly salient, such difficulties may motivate efforts to conceal communicative weakness or compensate for it in order to fit in. In this sense, camouflaging may function as a short-term adaptation to language-based social vulnerability. This interpretation is also consistent with broader evidence showing that social-pragmatic difficulties are associated with social and behavioural impairment (Saul et al., 2023).

At the same time, the present findings should not be interpreted as suggesting that camouflaging is necessarily beneficial. Recent research indicates that camouflaging can be emotionally costly. Reviews have shown that people camouflage in response to stigma, the need to belong, and pressure to appear socially typical, but that these efforts may contribute to burnout, identity strain, and poorer mental health (Klein et al., 2025; Zhuang et al., 2023). Qualitative work with autistic children and adolescents likewise suggests that camouflaging can be stressful, confusing, and exhausting (Howe et al., 2023). Although the present study did not directly measure anxiety or depression, the higher level of camouflaging in adolescents with SCD may indicate a subgroup that is carrying a heavier emotional burden in everyday interaction. That point is especially relevant to the present Research Topic, which emphasizes how language and emotional functioning intersect in mental health.

The finding that males reported more camouflaging than females should be interpreted cautiously, particularly given the relatively small number of female participants in the sample. The literature on gender differences in camouflaging is mixed. Some studies, especially in autistic samples, have reported greater camouflaging in females, whereas others have reported greater camouflaging in males or no significant difference (Hull et al., 2020; Milner et al., 2023; Ross et al., 2023). In the present sample, one possible explanation is that male adolescents may have experienced stronger social pressure to perform confidently in public or peer settings despite communication difficulty. Another possibility is that gendered patterns of socialization in the local context influenced how camouflaging strategies were expressed or reported. These interpretations remain tentative, and future research should investigate whether the observed gender difference reflects actual behavioural differences, reporting tendencies, or culture-specific expectations.

The subscale findings help clarify the nature of camouflaging in this sample. Higher compensation scores suggest that adolescents with SCD may actively develop strategies to work around social-communication difficulty, for example by preparing responses in advance or relying on learned conversational routines. Higher masking scores suggest greater efforts to hide communication difficulty or avoid drawing attention to it. Higher assimilation scores indicate stronger attempts to appear socially typical and blend into the interactional environment. Taken together, these findings support the view that camouflaging is multidimensional and may reflect different ways of managing the language demands of adolescence.

Several limitations should be acknowledged. First, the study was cross-sectional, so no conclusion can be drawn about the direction of association between SCD and camouflaging. Second, the sample was drawn from one region of Iran, which limits generalizability. Third, the CAT-Q was originally developed for autistic populations; although it is useful for capturing compensation, masking, and assimilation, it is not specific to SCD. Fourth, the study did not include direct measures of anxiety, depression, self-esteem, or exhaustion. As a result, the present study can speak to the potential emotional significance of camouflaging, but it cannot directly test mental health outcomes. Future studies should include mental health measures and, ideally, longitudinal designs to examine whether camouflaging predicts later emotional difficulties in adolescents with SCD. In addition, the relatively small number of female participants limits the strength of conclusions regarding gender differences and may have reduced the ability to detect more nuanced gender-related patterns in camouflaging.

Despite these limitations, the study contributes to an emerging literature that treats camouflaging as more than a behavioural style. In adolescents with SCD, camouflaging may represent a response to persistent language-pragmatic challenges in peer contexts and may also signal hidden emotional strain. Clinically, this suggests that intervention should focus not only on improving communication skills but also on reducing the need to camouflage by creating supportive, less stigmatizing social environments. Educational and psychological interventions that strengthen pragmatic communication, emotional safety, and peer understanding may help reduce the pressure to camouflage and thereby support better mental health outcomes.

## Data Availability

The raw data supporting the conclusions of this article will be made available by the authors, without undue reservation.
